# A cost effectiveness analysis of the preferred antidotes for acute paracetamol poisoning patients in Sri Lanka

**DOI:** 10.1186/1472-6904-12-6

**Published:** 2012-02-22

**Authors:** S M D K Ganga Senarathna, Shalini Sri Ranganathan, Nick Buckley, Rohini Fernandopulle

**Affiliations:** 1 Department of Pharmacology, Faculty of Medicine, University of Colombo, Colombo, Sri Lanka; 2 South Asian Clinical Toxicology Research Collaboration, Kandy, Sri Lanka; 3 Pharmacy Program, Department of Medical Education and Health Sciences, Faculty of Medical Sciences, University of Sri Jayewardenepura, Nugegoda , Sri Lanka; 4 Faculty of Medicine, University of New South Wales, Sydney, Australia

## Abstract

**Background:**

Acute paracetamol poisoning is a rapidly increasing problem in Sri Lanka. The antidotes are expensive and yet no health economic evaluation has been done on the therapy for acute paracetamol poisoning in the developing world. The aim of this study is to determine the cost effectiveness of using N-acetylcysteine over methionine in the management of acute paracetamol poisoning in Sri Lanka.

**Methods:**

Economic analysis was applied using public healthcare system payer perspective.

Costs were obtained from a series of patients admitted to the National Hospital of Sri Lanka with a history of acute paracetamol overdose. Evidence on effectiveness was obtained from a systematic review of the literature. Death due to hepatotoxicity was used as the primary outcome of interest. Analysis and development of decision tree models was done using Tree Age Pro 2008.

**Results:**

An affordable treatment threshold of Sri Lankan rupees 1,537,120/death prevented was set from the expected years of productive life gained and the average contribution to GDP. A cost-minimisation analysis was appropriate for patients presenting within 10 hours and methionine was the least costly antidote. For patients presenting 10-24 hours after poisoning, n-acetylcysteine was more effective and the incremental cost effectiveness ratio of Sri Lankan rupees 316,182/life saved was well under the threshold. One-way and multi-way sensitivity analysis also supported methionine for patients treated within 10 hours and n-acetylcysteine for patients treated within 10-24 hours as preferred antidotes.

**Conclusions:**

Post ingestion time is an important determinant of preferred antidotal therapy for acute paracetamol poisoning patients in Sri Lanka. Using n-acetylcysteine in all patients is not cost effective. On economic grounds, methionine should become the preferred antidote for Sri Lankan patients treated within 10 hours of the acute ingestion and n-acetylcysteine should continue to be given to patients treated within 10-24 hours.

## Background

Paracetamol is the most common cause of drug poisoning in the world [1] and the single most commonly taken drug in overdoses that lead to hospital presentation and admission [2].

Poisoning with paracetamol is an emerging problem in Sri Lanka with rapidly increasing admissions to the National Hospital of Sri Lanka (NHSL): from only 35 cases in 2003 to 515 cases in 2005 [3]. Paracetamol poisoning is one of the most expensive poisonings management in Sri Lanka [4,5]. The average cost of managing a patient with acute paracetamol poisoning was even higher than the average cost of managing a organophosphate poisoning patient; the most costly poisoning management at the Anuradhapura General Hospital in Sri Lanka [4,5]. However it is important to note that eighty percent of the current total cost of management of acute paracetamol poisoning is due to cost of antidotes unlike in organophosphate poisoning [5].

Even though paracetamol poisoning has been researched far more than other pharmaceutical poisonings, there is limited literature on the pharmaco-economics of treatment. So far no full economic evaluation on interventions on paracetamol poisoning has been carried out [6]. Two antidotes, N-acetylcysteine (NAC) and methionine, are available in Sri Lanka. NAC is the most expensive and also the most commonly used antidote. The evidence on effectiveness of antidotes used in acute paracetamol poisoning is weak [7]. However, on the basis of the current best available evidence NAC is considered to be more effective than methionine in the management of patients with acute paracetamol poisoning [7]. Considering that NAC is also more costly than providing methionine, it should be useful to determine the comparative cost effectiveness of the two treatments, and whether the additional benefit of NAC over methionine is worth the extra cost. Cost effectiveness analysis is based on the premise that it is the wider community interest which is paramount; therefore extra lives saved from use of a more expensive antidote have to be balanced against the extra costs involved in doing so. It is easy to make the management decision; if an intervention is dominant (i.e. the new intervention is less costly and yields higher benefit). But in situations where an intervention is not dominant, we have to find out the point at which the intervention is cost effective. The question of an intervention is cost-effective depends upon whether the relevant decision maker is willing and able to pay the additional costs to achieve the additional benefits that can be achieved by introducing the alternative program. The magnitude of this value will be controversial. In this analysis it was considered that the program is cost effective if it can gain a year of healthy life for less than a country's national income per person per capita gross national income [8].

The objective of the present cost effectiveness analysis is to determine the incremental cost effectiveness of NAC over methionine in the management of acute paracetamol poisoning patients (with suicidal intent) in Sri Lanka.

## Methods

### Structure of the decision tree model

The evidence on effectiveness of both antidotes; NAC and methionine depends on the post ingestion time. According to the best available evidence, all antidotes are much more effective if given within 10 hours of the acute ingestion [7].

**Figure 1:**
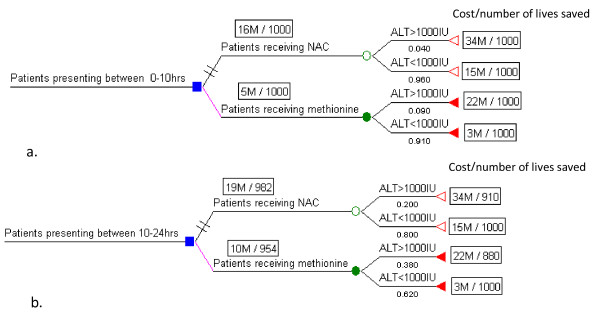
**The decision represented by the node (square) at the left, is between receiving antidote, NAC and methionine when a patients is presented for treatment within 10 hours (a) and 10-24 hours (b) of the acute ingestion of paracetamol**. Each chance event is shown as a solid circle and represent either the chance of developing liver failure (ALT > 1000 IU) or not developing liver failure. The numbers on each branch are probabilities and outcome is represented by cost [in million (M) rupees (LKR)] per number of lives saves (1000 hypothetical patients in each arm were considered) at the terminal node (rectangular). The probabilities and outcomes are explained in Table 1.

Therefore we constructed two decision tree models: for patients treated within 10 hours, and for patients treated within10 to 24 hours after acute ingestion of paracetamol (Figure 1). Both models compared use of methionine and NAC in patients where the risk was assessed using plasma paracetamol levels according to the Rumack-Matthew nomogram.

Acute paracetamol poisoning can result in fulminant hepatic failure which can lead to death. The most frequently applied definition for significant paracetamol hepatotoxicity is having liver transaminases (ALT or AST) > 1000 U/L at any time [9,10].

Therefore the decision tree model adopted death following hepatotoxicity (ALT or AST > 1000 U/L) as the final outcome measure (Figure 1).

### Population considered

The analysis was applied to patients admitted to the National Hospital of Sri Lanka (NHSL). The ethics approval was obtained from ethical review boards of the NHSL and the Faculty of Medicine, Colombo. In Sri Lanka healthcare is free at the point of delivery. Therefore the analysis was done from the public healthcare system payers' perspective. The time horizon was 2006 and the analysis was performed with reference to 1000 hypothetical patients in each arm.

### Data and assumptions on costs and effectiveness of antidotes

#### Costs

The direct costs linked to the treatment options were measured as the total cost of drug treatment and hospitalisation. The total cost of drug treatment includes the cost of NAC/methionine (obtained from the medical supplies division of Sri Lanka), cost of hospital stay (obtained from accounts branch of the NHSL and follow up of case series) and cost of person hours (obtained from accounts branch of the NHSL and follow up of case series). The cost of antidotal therapy was based on the usual NAC (IV NAC 300 mg/kg over 20.25 hours) and methionine regimen(2.5 g every four hours for four doses) used in Sri Lanka. The costs in LKR (Sri Lankan rupees) (100 LKR = 1 US dollar) of items included in the analysis are given in Additional file 1: annexure1.

#### Effectiveness data

Antidotal effectiveness data was obtained from the systematic review and meta-analysis on the interventions for paracetamol poisoning by Brok et al. 2002 and 2006 [6,7]. The meta analysis included randomised controlled trials(RCTs), quasi-RCTs, RCTs with volunteers and observational studies. According to the review, no RCTs were of high quality. Further, no RCT compared NAC with methionine or no treatment for the relevant time frame (within24 hours of the acute ingestion) for this analysis. The review provided an exploratory analysis on antidotes and gave pooled probabilities for developing hepatotoxicity and death following acute paracetamol poisoning in patients with plasma paracetamol levels above possible or probable risk line of the nomogram [10,11]. Summary of the studies included in the exploratory analysis are given in Additional file 2: annexure 2 [9-21].

#### Incremental cost effectiveness

The total costs and outcomes for each treatment arm were presented. The expected value of each management alternatives identified at the root. The incremental cost effectiveness ratio (ICER) for two alternative treatments was calculated (incremental analysis produces a summary measure of relative efficiency) by dividing the cost difference by the outcome difference.

$$\text{ICER}=\left(\text{Cost of NAC }-\text{ cost of methionine}\right)/\left(\text{Outcome for NAC }-\text{ outcome for methionine}\right)$$

When there was no difference in outcome between management alternatives, cost minimisation analysis was done and the least costly management alternative was chosen.

Development of decision tree models and the analysis were done using Tree Age Pro Excel healthcare 2008 software, Serial number GXT2J-2B6KK-3D3DV-G.

#### Discounting and sensitivity analysis

Acute paracetamol poisoning is an acute condition and costs and outcomes occur during a short span of time: two to ten days. Therefore costs or outcomes were not discounted to adjust for elapsed time between expenditure and outcome when ICERs are calculated. When treatment threshold value was calculated, future income of the study patients were considered and therefore it was discounted at the rate of 3.5% to bring it to the present value [22],

One-way-sensitivity analysis was done by increasing and decreasing costs and mortality by 50% and also by taking the upper and lower confidence interval of the probability on the systematic review estimates of mortality and hepatotoxicity with each antidote. Multi- ways sensitivity analysis was done to examine the worst combination of the single factors for both NAC and methionine in both time periods.

## Results

### Medical care cost

When a 60 kg patient is treated with NAC and does not develop hepatotoxicity, the cost to the healthcare system was 15,038 rupees. However if the patient develops hepatotoxicity, the cost rises to 34,329 rupees due to infusion of additional NAC and supportive care. The total cost for the methionine option was 2,839 rupees when patients do not develop hepatotoxicity and 22,481 when the patient develops hepatotoxicity. The breakdown on direct medical care costs are given in Additional file 1: annexure 1.

### Outcome data

The exploratory analysis of the systematic review pooled studies together and gave pooled probabilities as shown in Table 1[7]. The probability for death following the use of both antidotes was zero for patients treated within 10 hours of the acute ingestion. However, the 95% CI of the case-fatality had an upper CI of 0.5% for NAC and 2.4% for methionine.

**Table 1:** Probability for hepatotoxicity and death, when antidotes are administered within 0-10 and 10-24 hours from the acute ingestion

	Probability for AST/ALT > 1000 IU/l (n/N) [6,7]	Probability for death (n/N)	Note
**IV NAC 300 mg/kg over 20.25 hours**			

Within 10 hours	4% (13/315)	0^†^(0/13)	*
	(95% CI 2.5 to 7.0)	(95% CI 0 to 24)	
Within 10-24 hours	20% (67/322)	9%^‡ ^(6/67)	*
	(95% CI 17 to 26)	(95% CI 3.8 to 18)	

**Methionine 1 g 4 hourly 4 doses**			

Within 10 hours	9% (13/143)	0^†^	#
	(95% CI 4.5 to 15)	(95% CI 0 to 24)	
Within 10-24 hours	38%(17/41)	12%^Π^(2/17)	#
	(95% CI 26.3 to 57.9)	(95% CI 2 to 36)	

* Probabilities were derived by pooling results of Prescott et al. 1979, Smilkstein et al. 1988, Burkhart et al. 1995, Buckley et al. 1999, Parker et al. 1990, Smilkstein et al. 1991, Woo et al. 2000, Ayonrinde et al. 2005 and Kerr et al. 2005 [9-17]. # Probabilities were derived by pooling results of Prescott et al. 1979, Crome et al. 1976, Hamlyn et al. 1981, Prescott et al. 1976 and Vale et al. 1981 [9,18-21]. † No patient treated (NAC/methionine) within 10 hours died due to hepatototoxicity, therefore the probability of death due to hepatotoxicity was zero

### Threshold value

The mean per capita income for a Sri Lankan in 2006 was LKR 6,463/month [23]. The average age for the case series of patients was 20 years and the average number of working years for a Sri Lankan is 55 years which is the retirement age. When patients poisoned with paracetamol dies at the age of 20, the society will lose average earning at the rate of percapita income per month up to the retirement age. Therefore the discounted present value of a life saved at the age of 20 would be LKR 1,537,120

(Discounted present value = ∑^35-1 ^77556_n _(1-0.035)^n^) and this value was used as the treatment threshold value to prevent a death due to acute paracetamol poisoning in Sri Lanka. Therefore the study considered that it is cost effective to prevent an additional death by NAC at a cost of ≤ LKR 1,537,120.

### Baseline results

The decision tree models with expected outcomes for patients presenting within 10 and 10-24 hours is given in Figure 1.

The incremental cost effectiveness ratios reveal how much it would cost to prevent an extra death by shifting to the more costly antidote (NAC) from the cheaper alternative antidote (methionine). When treated within 10 hours of the acute ingestion, the incremental cost for treatment with NAC over methionine came to LKR 11,588,680, but outcome resulted from alternative interventions were similar. Therefore base line ICER was not calculated for this group and cost minimisation analysis was done (Table 2).

**Table 2:** Incremental cost per life saved for baseline data and following one way sensitivity analysis (Number of lives saved as the final outcome measure)

	Incremental cost per life saved(LKR/Life saved)	
	
	0-10 hrs	10-24 hrs
**Baseline results**	Not applicable^‡^	316,182

**One way sensitivity analysis results**		

***Increasing by 50%***		

NAC deaths	Not applicable^‡ ^	469,173
NAC cost	Not applicable^‡ ^	565,805
Methionine deaths	Not applicable^‡ ^	173,147
Methionine cost	Not applicable^‡ ^	265,107

***Decreasing by 50%***		

NAC deaths	Not applicable^‡ ^	238,432
NAC cost	Not applicable^‡ ^	55,312
Methionine deaths	Not applicable^‡ ^	1,818,046
Methionine cost	Not applicable^‡ ^	366,486

***Upper confidence interval(CI)***		

NAC deaths	Dominance for methionine ^† ^	909,023
NAC hepatotoxicity	Not applicable^‡ ^	445,229
Methionine deaths	5,365,130	73,456
Methionine hepatotoxicity	Not applicable^‡ ^	94,349

***Lower confidence interval(CI)***		

NAC deaths	Not applicable^‡ ^	229,648
NAC hepatotoxicity	Not applicable^‡^	268,907
Methionine deaths	Not applicable^‡ ^	Dominance for methionine ^† ^
Methionine hepatotoxicity	Not applicable	836,480

^‡ ^The outcome resulted from alternative interventions were similar therefore cost effectiveness ratios were not calculated.

^† ^Methionine shows strong dominance for a decision as the incremental effectiveness was lower while the incremental cost is higher for NAC (methionine saves more lives at a lower cost)

Cost effectiveness analysis was done for patients treated within 10 to 24 hours, the ICER for this group of patients was LKR 316,182 per life saved (below the threshold value) where the incremental cost was LKR 8,726,620 and additional lives saved by NAC was 28 (Table 2).

### Sensitivity analysis

#### One way sensitivity analysis

Incremental cost effectiveness ratios for one way sensitivity analysis, taking mortality as outcome measure are given in Table 2.

#### Multi-way-sensitivity analysis

According to the base-case analysis, methionine was the lease costly antidote for patients treated within 10 hours and NAC had an ICER below the threshold value for patients treated within 10 to 24 hours after ingestion. The robustness of this decision was further tested by assessing the worse case for methionine when given within 10 hours and the worse case for NAC when given 10-24 hours in a multi-way sensitivity analysis.

Worse case analysis for methionine within 10 hours gave an ICER of LKR 2,718,784 for NAC. Worse case analysis for NAC given within 10-24 hours showed dominance for methionine where methionine saved 42 more lives at a lower cost.

## Discussion

The most important factor influencing total cost in the management of acute paracetamol poisoning in Sri Lanka is the antidotal therapy. This cost pattern would also be observed in other developing country settings where hospital stay and human resources are cheaper. Economic modeling should inform the most cost effective way of using these two antidotes which can reduce the total cost of management in developing country settings.

The most suitable economic analysis for patients treated within 10 hours of the acute ingestion is the cost minimization analysis, as the evidence suggests the probability of death is zero for patients treated with either antidote. With this premise, methionine is clearly the least costly alternative for this group of patients. One-way sensitivity analysis and multi-way sensitivity analysis calculated ICERs (on instances where there is an outcome difference) were above our pre-defined threshold for an acceptable cost per life saved by NAC. Therefore, methionine is the antidote of choice for patients treated within 10 hours.

The ICER for NAC for patients presenting within 10-24 hours was LKR 316,182 for a death prevented. This is a value much lower than the treatment threshold value and suggests the use of NAC in preference to methionine is very cost-effective. One way sensitivity analysis in all instances had ICERs lower than the threshold value except when the lower confidence interval for death following methionine was considered where it showed dominance for methionine.

Worse case analysis for NAC also indicated dominance for methionine. Therefore the conclusion ranged from dominance for methionine to NAC being cost effective. However in most of the sensitivity analysis and in the base case analysis the ICER for NAC was much lower than the threshold value. Therefore use of NAC for patients presenting within 10-24 hours of the acute ingestion appears to be a cost effective option in Sri Lanka.

The effectiveness data for the decision tree was based on the systematic review and meta analysis by Brok et al. 2006. The meta-analysis referred to 10 studies to produce estimates of effectiveness in respect to these two antidotes; five studies for NAC, four studies for methionine and one study on both antidotes. According to the levels of evidence and grade of recommendation proposed by Cook et al. 1992 [24], studies used for methionine were in grade B(1), grade C(1) and grade D(2), studies used for effectiveness of NAC were in grade B(1), Grade D(2)and grade E(2) and the study on both NAC and methionine was grade D quality.

The meta-analysis did not use any studies which provided grade A recommendations and eight out of 10 studies were in grade D or worse. An RCT directly comparing these two antidotes would provide much better effectiveness data. Such a trial might be worthwhile and should be strongly supported. The trial could not easily be blinded due to the different routes of administration. However, the outcome measures are largely objective and there is a low likelihood of bias.

## Conclusions

Post ingestion time is an important determinant of preferred antidotal therapy for acute paracetamol poisoning patients in Sri Lanka. Using N-acetylcysteine in all patients is not cost effective.

If policy makers are wishing to utilise economic evaluations to improve decision making, the findings from this study suggest that, within the first 10 hours, the use of methionine may be more cost-effective than NAC for paracetamol poisoning in Sri Lanka. This would potentially more than halve the total expenditure on this increasingly common poisoning. N-acetylcysteine should continue to be given to patients treated within 10-24 hours.

### Limitations

The study recommends the use of methionine for patients treated within 10 hours of the acute ingestion. There is some literature suggestive of methionine induce adverse effects such as nausea and vomiting. However our prospective case series didn't identify such incidences even though 55 patients received methionine compared 68 of IV NAC. Two patients received both antidotes owing to unavailability of IV NAC at the time of patient admission [3].

## Abbreviations

GDP: Gross domestic product; NHSL: National Hospital of Sri Lanka; NAC: N-acetylcysteine; ALT: Serum alanine aminotransferase; AST: Serum aspartate aminotransferase; LKR: Sri Lankan rupees; RCTs: Randomised controlled trials; ICER: Incremental cost effectiveness ratio.

## Competing interests

The authors declare that they have no competing interests.

## Authors' contributions

SMDKGS participated in the design and planning of the study, drafted the study proposal, collected patient information, conducted the economic evaluation and interpreted results, wrote the first draft and took part in revising the paper and finalizing the paper. SSR participated in the design and planning of the study, edited and revised the study proposal, edited the paper and took part in revising the paper and approved the final version. NB participated in the design and planning of the study, edited and revised the study proposal, edited the paper and took part in revising the paper and approved the final version. RF participated in the design and planning of the study, edited and revised the study proposal, edited the paper and took part in revising the paper and approved the final version. All authors read and approved the final manuscript.

### Study design

Cost effectiveness analysis based on exploratory analysis of a systematic review

## Pre-publication history

The pre-publication history for this paper can be accessed here:

http://www.biomedcentral.com/1472-6904/12/6/prepub

## Supplementary Material

Additional file 1 **Annexure 1**. Cost per patient (body weight: 60 kg) for different treatment alternatives. Click here for file

Additional file 2 **Annexure 2**. Summary of the studies included in the exploratory analysis of the systematic review [9-21]. Click here for file
