# Supplementary material for: A cost effectiveness analysis of the preferred antidotes for acute paracetamol poisoning patients in Sri Lanka
Source: BMC Clin Pharmacol. 2012 Feb 22;12:6. doi: 10.1186/1472-6904-12-6 (PMC3350452; doi:10.1186/1472-6904-12-6)
Supplement: Additional file 1 — Annexure 1. Cost per patient (body weight: 60 kg) for different treatment alternatives. [file 1472-6904-12-6-S1.DOC]

**Annexure 1. Cost per patient (body weight: 60kg) for different treatment alternatives**

| **Cost for treatment alternatives** | **Cost in LKR** |
| --- | --- |
| NAC vials | 14,400 |
| 5% dextrose | 71 |
| Doctor, nurse and other person hour cost | 393 |
| HBDs/hotel cost^†^ | 174 |
| **Total cost for NAC <1000IU ALT** | **15,038** |
| NAC vials | 14,400 |
| 5% dextrose | 71 |
| Lactulose 30ml QID for 7 days | 840 |
| Monitor blood glucose for 7 days | 770 |
| NAC 100mg/kg for 16 hours + 1000ml 5%dextrose ( 3 infusions ) | 16,926 |
| Doctor, nurse and other person hour cost | 712 |
| HBDs / hotel cost^†^ | 610 |
| **Total cost for NAC > 1000IU ALT ( Acute liver failure ;supportive care)** | **34,329** |
| Methionine tablets | 2,291 |
| Doctor, nurse and other person hour cost | 374 |
| HBDs/ hotel cost^†^ | 174 |
| **Total cost for Methionine <1000IU ALT** | **2,839** |
| Methionine tablets | 2,291 |
| Lactulose 30ml QID for 7 days | 840 |
| Monitor blood glucose for 7 days | 770 |
| NAC 100mg/kg for 16 hours + 1000ml 5%dextrose (3 infusions) | 16,926 |
| Doctor, nurse and other person hour cost | 693 |
| HBDs /hotel cost^†^ | 610 |
| **Total cost for Methionine > 1000IU ALT (Acute liver failure ;supportive care)** | **22,130** |

^†^ Hospital bed day (HBD) costs in our study include only the regular costs incurred during the hospital stay excluding costs of pharmaceuticals and other variable costs (therefore the HBD cost is also referred to as the hotel cost).
