# Supplementary material for: A cost effectiveness analysis of the preferred antidotes for acute paracetamol poisoning patients in Sri Lanka
Source: BMC Clin Pharmacol. 2012 Feb 22;12:6. doi: 10.1186/1472-6904-12-6 (PMC3350452; doi:10.1186/1472-6904-12-6)
Supplement: Additional file 2 — Annexure 2. Summary of the studies included in the exploratory analysis of the systematic review [9-21]. [file 1472-6904-12-6-S2.DOC]

Annexure 2: Summary of the studies included in the exploratory analysis of the systematic review [10-19]

| **Study** | **Type** | **No of patients** | **Inclusion criteria** | **Intervention** | **Comparison group** | **Outcome** | **Grade** |
| --- | --- | --- | --- | --- | --- | --- | --- |
| **Buckley et al 1999a** | Prospective observational study | 86 | Paracetamol concentration > probable risk line / ingested dose >125 mg/kg if serum concentration is not available within 8hrs / > possible risk line if the calculated t1/2 is >4 hrs | IV NAC 300mg/kg over 20.25hrs | Historical patients treated at other units given other doses of NAC (oral or IV) | Mortality | D |
| **Parker et al 1990** | Prospective observational study | 20 | Paracetamol  concentration > probable risk line | IV NAC (300 mg/kg in 20 hours). Gastric lavage in five patients. | None | Mortality, AST, bilirubin, INR | E |
| **Prescott et al 1979** | Retrospective observational study | 217 | Paracetamol concentration > probable risk line | Gp1: IV NAC (300 mg/kg for 20 hours)  Gp 2: either IV methionine (total dose: 20 g) or cysteamine (total dose: 3.6 g). | Historical patients on supportive treatment | Mortality, ALT, bilirubin, INR | D |
| **Smilkstein et al 1991** | Prospective observational study | 179 | Paracetamol concentration > possible risk line | Loading dose of 140 mg/kg IV NAC followed by 12 doses  of 70 mg/kg | Historical patients from other studies given oral or IV NAC | Mortality, AST, ALT | D |
| **Ayonrinde et al 2005** | Retrospective observational study | 99 | Paracetamol concentration > probable risk line | IV NAC 300mg/kg 20.25hrs | No control group | AST | E |
| **Kerr et al 2005** | RCT  ( randomised slips in a closed box) | Gp 1 = 71  Gp 2 =109 | On paracetamol concentration & time of acute ingestion | Gp 1 : IV NAC 150 mg/kg over 60 min  ( total dose 300mg/kg) | Gp 2 : IV NAC 150 mg/kg over 15 min  ( total dose 300mg/kg) | Adverse events ,ALT | B |
| **Hamlyn**  **et al 1981** | RCT  (sealed envelopes, balanced block randomisation stratifying for age) | 40 | Paracetamol concentration > probable risk line | Gp 1: Supportive T. & cysteamine in Newcastle. Gp 2: Supportive T. & cysteamine in London.  Gp 3: Supportive T. & methionine in Newcastle. Gp 4: Supportive therapy & methionine in London | Supportive therapy only in Newcastle | AST, bilirubin, INR, liver biopsy findings | B |
| **Crome**  **et al 1976** | Retrospective observational study | 30 | Paracetamol concentration > probable risk line | Methionine 2.5g orally every four hours (Total dose of 10g ) | Patients given supportive treatment at other units | AST , bilirubin | C |
| **Prescott**  **et al 1976** | Retrospective observational study | 121 | Paracetamol concentration < possible risk-line | Gp1: Cysteamine given IV loading dose (2.0 g) followed by an infusion of 1.6 g for 20 hours  Gp 2: Methionine given IV an loading dose (2.0 g) followed by 15.0 g  infused over 20 hours | Historical patients with no antidote treatment. | AST,ALT bilirubin, ,INR | D |
| **Vale et al 1981** | Retrospective observational study. | 132 | Paracetamol concentration > a risk-line from 220 mg/l at four hours and 70 mg/l at 12 hours | Four doses of 2.5 gram of oral methionine over 12 hours. | Historical patients with no antidote treatment | Mortality, hepatotoxicity and serum creatinine. | D |
